# The Role of Mean Platelet Volume as a Predictor of Mortality in Critically Ill Patients: A Systematic Review and Meta-Analysis

**DOI:** 10.1155/2016/4370834

**Published:** 2016-02-04

**Authors:** Pattraporn Tajarernmuang, Arintaya Phrommintikul, Atikun Limsukon, Chaicharn Pothirat, Kaweesak Chittawatanarat

**Affiliations:** ^1^Department of Internal Medicine, Faculty of Medicine, Chiang Mai University, Chiang Mai 50200, Thailand; ^2^Department of Surgery, Faculty of Medicine, Chiang Mai University, Chiang Mai 50200, Thailand

## Abstract

*Background*. An increase in the mean platelet volume (MPV) has been proposed as a novel prognostic indicator in critically ill patients.* Objective*. We conducted a systematic review and meta-analysis to determine whether there is an association between MPV and mortality in critically ill patients.* Methods*. We did electronic search in Medline, Scopus, and Embase up to November 2015.* Results*. Eleven observational studies, involving 3724 patients, were included. The values of initial MPV in nonsurvivors and survivors were not different, with the mean difference with 95% confident interval (95% CI) being 0.17 (95% CI: −0.04, 0.38; *p* = 0.112). However, after small sample studies were excluded in sensitivity analysis, the pooling mean difference of MPV was 0.32 (95% CI: 0.04, 0.60; *p* = 0.03). In addition, the MPV was observed to be significantly higher in nonsurvivor groups after the third day of admission. On the subgroup analysis, although patient types (sepsis or mixed ICU) and study type (prospective or retrospective study) did not show any significant difference between groups, the difference of MPV was significantly difference on the unit which had mortality up to 30%.* Conclusions*. Initial values of MPV might not be used as a prognostic marker of mortality in critically ill patients. Subsequent values of MPV after the 3rd day and the lower mortality rate unit might be useful. However, the heterogeneity between studies is high.

## 1. Introduction

Critically ill patients are defined as those patients who have physiologic instability, which may lead to morbidity and mortality within a short period of time. These patients need intensive monitoring of organs functions such as functions related to the cardiovascular, the respiratory, and the neurological systems. Most critically ill patients are transferred to the intensive care unit (ICU) for close monitoring. There are approximately 4 million ICU admissions per year in the United States with average mortality rate reported ranging from 8% to 19%, or about 500,000 deaths annually, in which sepsis is one of the leading causes of admission and mortality. As we all know, sepsis is an inflammatory response of host to infection. Deterioration of sepsis leads to severe sepsis and septic shock, which has a high mortality rate. Approximately 150,000 patients die annually in Europe and more than 200,000 annually in the United States due to severe sepsis [1, 2].

It is not uncommon that the hematologic system is affected by potent inflammation in critically ill patients. The hematologic profiles, including hematocrit, white blood cell count, and platelet count, are used in many widely accepted prognostic prediction scores such as sequential organ failure assessment (SOFA) score, multiple organ dysfunction score (MODS), and logistic organ dysfunction score (LODS) [3–5].

Currently, many physicians have shown interest in platelet indices, particularly the platelet volume, because it may reflect the platelet function better than the platelet count itself. Nowadays, platelet volume is usually reported in routine complete blood count results. The mean platelet volume (MPV) reflects the platelet size. Elevation of MPV is suggestive of increasing platelet production and activation. Larger platelets also contain more granules and prothrombotic materials [6, 7]. Many clinical studies have shown an association between high MPV and thromboembolic events, also with adverse cardiovascular and cerebrovascular outcomes. A recent meta-analysis showed the prognostic value of MPV in coronary artery diseases (CAD) [8–11].

However, in non-CAD critical illnesses, the correlation between high MPV and poor prognosis is still unclear. Many cohort studies showed an association between MPV and poor outcome in critically ill patients, but the same was not the case in other studies.

The primary objective of this analysis is to determine the association between MPV and mortality in non-CAD critically ill patients. The secondary objective is to determine the association between MPV and mortality in the subgroup of patients with sepsis.

## 2. Materials and Methods

### 2.1. Search Strategies

We searched for published and unpublished studies from Medline, Scopus and Embase databases with restrictions in terms of neither the published year nor the language. The MeSH Terms included “mean platelet volume” or “platelet index” or “platelet size” and “sepsis” or “septic shock” or “critically-ill” or “intensive care.” The search strategies of each database are described in the Supplemental Appendix A in Supplementary Material available online at http://dx.doi.org/10.1155/2016/4370834. Hand searching was also performed in the reference lists of the included studies to identify additional eligible studies. Additionally, one author (Pattraporn Tajarernmuang) attempted to contact a corresponding author regarding an unpublished study but failed to obtain the data.

### 2.2. Selection of Studies

All English abstract published studies were selected if they provided the data regarding MPV in intensive care units or sepsis patients and if mortality was the outcome of interest. The included studies needed to have sufficient data for pooling, that is, mean and standard deviation (SD) or median and interquartile range (IQR) of MPV between the groups of surviving and nonsurviving patients and the number of patients in each group. Studies were excluded if there were insufficient data and if there was no response from the authors.

### 2.3. Data Extraction

The data of eligible studies were independently extracted by two authors (Pattraporn Tajarernmuang and Arintaya Phrommintikul). The baseline characteristics of the included studies were, first, the author's name, publication year, study design, number of patients in survival and nonsurvival groups, age, sex, APACHE II score (if provided), and SOFA score (if provided). The parameters of interest were mean (±SD) of MPV and daily MPV in each group (if provided). If results were expressed as median and IQR, the mean (±SD) of MPV was acquired by contacting the corresponding authors or by converting using the formula, as suggested by Wan et al. [12]. Disagreements, if any, were resolved after discussion.

### 2.4. Risk of Bias Assessment

The quality and risk of bias of each study, using the Newcastle and Ottawa risk of bias criteria [13], were assessed independently by two reviewers (Pattraporn Tajarernmuang and Arintaya Phrommintikul). There were three domains to assess, that is, the selection of the study group, the comparability of the groups, and the ascertainment of outcome. The total score was 9; the higher the score, the lower the risk of bias. Disagreements, if any, were discussed, and then the conclusions were made.

### 2.5. Statistical Analysis

The mean values of MPV between the survival and the nonsurvival groups were estimated and pooled using the unstandardized mean difference (USMD). Heterogeneity between the studies was assessed using *Q* statistic and *I* square, which could be obtained from the inclusion and the exclusion criteria of each study, the mortality rate in each study, the level of hospital care, and the method and the time of the MPV measurement. If heterogeneity was present (*I* ^2^ > 25% or *p* < 0.10), the Dersimonian and Laird method (random effects models) was applied for all comparisons. Otherwise, if no heterogeneity was observed, the inverse variance method (fixed effects model) was used instead [14, 15]. Publication bias was examined using the Egger test and the funnel plot [16]. All the statistical analyses were carried out by using the STATA software, version 12. A *p* value < 0.05 was considered statistically significant for the two-sided test.

## 3. Results

**Figure 1:**
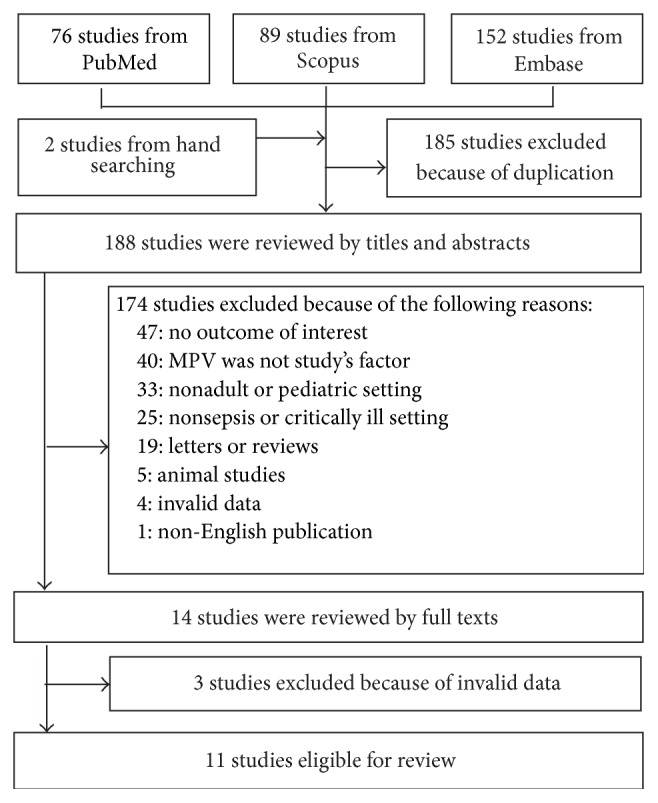
The flow chart of the study selection.

Three hundred and nineteen publications were identified: 76 from Medline, 89 from Scopus databases, 152 from Embase, and 2 from hand searching (last update on 4 November 2015). A total of 185 publications were duplicated and, thus, were excluded. A total of 188 titles and abstracts were screened and 174 articles were excluded. Fourteen publications were reviewed thoroughly, and 3 of these were excluded due to invalid data. Finally, 11 cohort studies, involving 3724 subjects, were included [17–27]. Reasons for exclusion from the studies are shown in Figure 1. Two of the excluded articles were abstracts from poster presentation, the full publication of which could not be found, and the data from the abstract were not sufficient for pooling [28].

The characteristics of the study subjects are shown in Table 1. Most studies were of cohort type; however, eight of them were retrospective [17, 19–23, 25]. MPV in subsequent days, which was available in five studies, is shown in Supplemental Table 1. The risk of bias assessment of ten eligible studies, using the qualitative assessment form, is presented in Supplemental Table 2. The total scores were found to range from 5 to 8.

**Figure 2:**
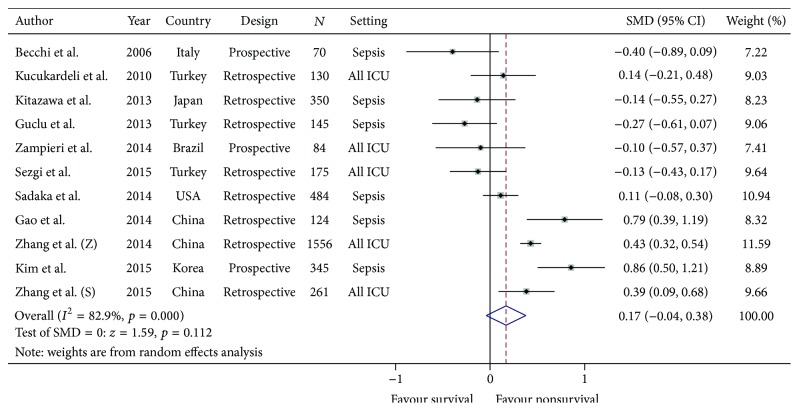
The pooled mean differences of the mean platelet volume between critically ill nonsurvivors and survivors.

Eight of eleven studies reported using mean MPV and SD of survivors and nonsurvivors in septic patients [17, 18, 20, 21, 23, 25–27]. The other three studies reported using median MPV and IQR in each group [19, 22, 24]. After our attempts to establish contact with the authors, we could obtain raw data from one author, from which we could analyze the mean and the SD [24]. For the other two studies, we converted the median and the IQR to mean and SD using the formula suggested by Wan et al. [12]. All the data were pooled and categorized into survival group and nonsurvival group. Six of eleven studies showed higher MPV in the nonsurvival group than in the survival group at the time of enrollment [18, 19, 22, 24–26]. The pooled mean difference of MPV between nonsurvivors and survivors was 0.17 (95% CI: −0.04, 0.38); however, there was marked heterogeneity by the random effect model (Chi square = 58.47, degree of freedom: 10, *I* ^2^: 82.9%, *p* < 0.001). See Figure 2.

The possible sources of heterogeneity were explored by age, sex, APACHE II, SOFA score study design, ICU setting, and mortality rate using metaregression analysis, and none of those factors was found to be the reason for the heterogeneity (Supplemental Table 3). The subgroup analysis of septic patients also showed no difference between survivors and nonsurvivors, with a mean difference of 0.17 (95% CI: −0.21, 0.55) (Table 2 and Supplemental Figure 1). However, the heterogeneity of this pooling was also high, as shown in Table 2. Although the tendency of MPV difference between survival and nonsurvival in retrospective study reports was higher than prospective study reports, the heterogeneity of this pooling was high (Table 2 and Supplemental Figure 2). In studies with mortality rate higher than 30%, the mean MPV also tends to be higher in nonsurvivors than in survivors, though not statistically significant (Table 2 and Supplemental Figure 3). In the sensitivity analysis, the heterogeneity studies were excluded by their possible heterogeneity characters and demonstrated in Table 3. Although the heterogeneity was high, the pooling mean of MPV was significantly higher in nonsurvivors after the small sample studies were excluded. The mean MPV difference was 0.24 (95% CI: 0.02 to 0.03) (Table 3 and Supplemental Figure 4).

**Figure 3:**
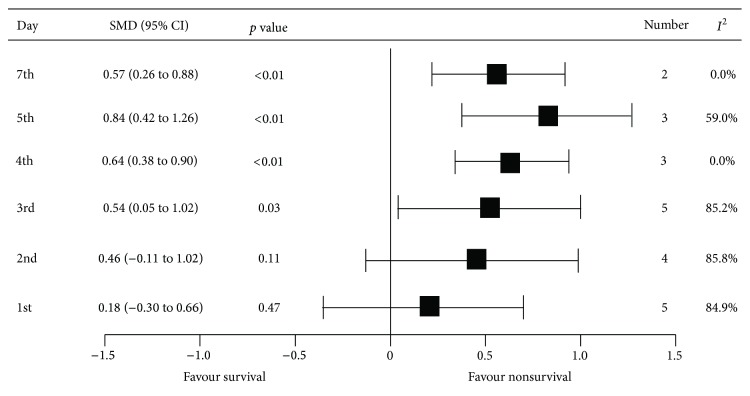
The summary of pooled mean differences of mean platelet volume on the observed day after admission between nonsurvivors and survivors.

The subsequent MPV values were also evaluated. Significant differences in MPV between nonsurvivors and survivors were observed on the third day after admission. The mean MPV differences on the 3rd, 4th, 5th, and 7th days were 0.54 (95% CI: 0.05, 1.02), 0.64 (95% CI: 0.38, 0.90), 0.84 (95% CI: 0.42, 1.26), and 0.57 (95% CI: 0.26, 0.88), respectively (Table 2, Figure 3, and Supplemental Figures 5–10).

The funnel plot showed asymmetry, which could be from either heterogeneity of studies or publication bias; interpretation by smaller studies showing statistical significance was not included (Supplemental Figure 11). However, Egger's test did not suggest small study effect (coefficient = 0.50, *p* value = 0.197).

## 4. Discussion

To our knowledge, this is the first systematic review and meta-analysis that assesses the prognostic value of MPV in noncardiac critically ill patients. There were eleven studies, including 3724 participants, totally, and the examination was for the association of MPV and mortality in critically ill patients. We found no significant correlation between initial MPV and hospital death. Subgroup analysis by sepsis, ethnicity, and mortality rate did not show any significant difference, as well.

However, we found that the gradual increase in MPV after a few days of admission was associated with increased hospital mortality (Supplemental Table 1). Increasing of MPV on subsequent days after admission in nonsurvivors was reported in five publications [18, 20, 22, 24, 26]. Two prospective studies demonstrated significant correlation between increased MPV and short-term mortality [24, 26]. Another study showed that the mean MPV level at the time of discharge was higher than the initial MPV level in nonsurvivors, while it was found to be decreasing in the survival group [23]. Therefore, it is evident that sequential monitoring of changes in MPV could be more important than single measurement.

Platelets play an important role in thrombogenesis [29]. The correlation between platelet activation and adverse clinical outcome of vascular diseases including coronary artery disease (CAD), stroke, and venous thromboembolism has been established. The mechanism of alteration of platelet function in sepsis is still unclear. The shape of platelets changes from discoid to spherical with pseudopodia during activation. MPV reflects the average size of platelets. Young platelets are larger than old platelets. Increased number of young platelets indicates increased platelet production due to overconsumption induced by inflammation. Larger platelets are functionally, metabolically, and enzymatically more active than smaller ones. They contain more intracellular thromboxane A2 and increased expression of procoagulant surface proteins such as p-selectin and glycoprotein IIIa, causing greater prothrombotic potential. Moreover, platelet-neutrophil interactions and platelet-endothelial interactions facilitate a variety of immune activation instances [6, 7, 30, 31].

MPV is available in laboratories worldwide; it is simple and cost-effective enough to be used as one of the prognostic markers in critically ill patients. In 1983, Van der Lelie and Von dem Borne demonstrated the existence of higher MPV in invasive infection than in localized infection and that the MPV value returned to normal when the disease became under control [32].

Our study showed the prognostic potential of the MPV trend to be better in less severe groups than in more severe groups (Table 2). MPV may be a useful predictor of prognosis when the coagulation system and the platelets are still strongly activated in early sepsis but not when coagulation factors and platelets are depleted in late or severe sepsis. Zampieri et al. showed that adding MPV to previous prognostic marker tools such as SAP-3 could increase the prognostic capability [24].

The limitation of our study was that most of the included studies were retrospective. The baseline MPV values of all the studies were different; consequently, finding the cut-point MPV value to predict mortality was inconceivable. This was supposed to be from different MPV measurement methods; four of the ten studies used the electrical impedance method, three of the ten studies used the optical mean method, and another three were not mentioned. The difference in the methods of measurement affected the MPV values [33]. The use of different anticoagulants also affected the MPV values [34, 35]; only 5 of the 11 publications reported using EDTA as the anticoagulant [17, 18, 20, 24, 26]. The time from venipuncture to measurement could also have affected the MPV value; platelets become larger over time after obtaining blood [36]. EDTA can cause the swelling of platelets, so the analysis should be done within 1 hour when EDTA is used [34]. There were many conditions that might have influenced MPV such as antiplatelet condition, smoking, preexisting hematologic diseases, liver diseases, malignancy, various inflammatory diseases, or any recent transfusions [37–45]. The differences in the exclusion criteria in each study could also have caused some degree of heterogeneity. Nevertheless, such heterogeneity among critically ill patients is always found in real world clinical practice.

## 5. Conclusions

Initial high MPV might not be used as a prognostic marker of mortality in critically ill patients; however, subsequent MPV might be useful. Future studies with well-designed prospective cohort are warranted.

## Supplementary Material

Search terms and search strategy used for PubMed, Scopus and Embase (“mean platelet volume” OR “platelet ind∗” OR “platelet size∗”) AND (sepsis OR “septic shock” OR “intensive care” OR “critically ill∗”).

## Figures and Tables

**Table 1:** Characteristics of the study.

Authors	Year	Country	Study design	Setting	Nonsurvivors						Survivors					
					Number	Age (year)	Male (%)	MPV (fL)	APACHE II	SOFA	Number	Age (year)	Male (%)	MPV (fL)	APACHE II	SOFA
Becchi et al. [18]	2006	Italy	Prospective	Sepsis	44	NA	NA	10.0 ± 1.7	NA	NA	26	NA	NA	10.5 ± 0.9	NA	NA
Kucukardali et al. [17]	2010	Turkey	Retrospective	All	62	76.5 ± 11.1	NA	8.5 ± 1.5	17.7 ± 5.0	NA	68	65.7 ± 21.6	NA	8.3 ± 1.2	13.59 ± 5.81	NA
Guclu et al. [19]	2013	Turkey	Retrospective	Sepsis	94	60.7 ± 18.5	55 (58.5)	7.0 (7.0, 9.0)^*∗*^	NA	NA	51	66.5 ± 18.5	29 (56.9)	8.0 (7.0, 9.0)^*∗*^	NA	NA
Kitazawa et al. [20]	2013	Japan	Retrospective	Sepsis	25	72.3 ± 11.3	17 (68.0)	7.5 ± 1.1	NA	NA	325	66.8 ± 15.7	168 (52.0)	7.6 ± 1.0	NA	NA
Sadaka et al. [21]	2014	USA	Retrospective	Sepsis	170	70.0 ± 14.0	NA	10.6 ± 0.9	27 ± 9.0	11.3 ± 2.9	314	66 ± 15	NA	10.5 ± 0.9	23.0 ± 7.0	9.8 ± 2.8
Zampieri et al. [24]	2014	Brazil	Prospective	All	24	50.4 ± 19.5	14 (58.0)	10.8 (10.1, 11.2)^*∗*^	NA	8.4 ± 4.6	60	50.3 ± 16.4	31 (52.0)	10.9 (10.2, 11.7)^*∗*^	NA	4.3 ± 3.7
Sezgi et al. [23]	2015	Turkey	Retrospective	All	95	69.0 ± 14.3	60 (73.2)	8.8 ± 2.3	28.1 ± 9.6	NA	80	62.4 ± 15.2	36 (45.0)	9.1 ± 2.4	21.1 ± 8.8	NA
Zhang et al. (Z) [25]	2014	China	Retrospective	All	443	63.1 ± 20.0	307 (69.3)	11.1 ± 1.4	NA	NA	1113	60.7 ± 19.0	714 (64.1)	10.5 ± 1.4	NA	NA
Gao et al. [22]	2014	China	Retrospective	Sepsis	88	61.7 ± 17.9	NA	11.2 (10.5, 12.5)^*∗*^	35 (27, 37.5)^*∗*^	NA	36	61.2 ± 20.6	NA	10.3 (9.7, 11.0)^*∗*^	30 (28, 33)^*∗*^	NA
Kim et al. [26]	2015	Korea	Prospective	Sepsis	35	68.9 ± 13.0	25 (71.0)	9.5 ± 1.7	25.9 ± 6.8	11.1 ± 3.0	310	63.7 ± 15.9	144 (46.5)	8.5 ± 1.1	16.5 ± 6.6	7.7 ± 2.6
Zhang et al. (S) [27]	2015	China	Retrospective	All	57	64 (53, 76)^*∗*^	35 (61.4)	15.8 ± 4.3	18 (13, 25.5)^*∗*^	9 (7, 14)^*∗*^	204	52 (43, 61)^*∗*^	156 (76.5)	12.8 ± 8.5	11 (8, 16)^*∗*^	5 (3, 8)^*∗*^

*Note*. The data presented are age, MPV, APACHE II, and SOFA shown as mean ± SD, ^*∗*^median (IQR), and *n* (%); NA: not available; Number: number of patients in the study; MPV: mean platelet volume; fL: femtoliter; APACHE II: acute physiologic and chronic health evaluation II score; SOFA: sepsis and organ failure assessment score. All the data are a mix of all the ICU population.

**Table 2:** Heterogeneity of pool mean differences of MPV in all studies and subgroup of patient type, study type, and mortality rate.

	Number of studies	*I* square (%)	SMD	(95% confidence interval)	*p* value
All studies	11	82.9	0.169	(−0.040 to 0.378)	0.112
Patient types					
Sepsis	6	86.7	0.166	(−0.215 to 0.546)	0.393
All ICU	5	75.9	0.169	(−0.040 to 0.378)	0.160
Study type					
Prospective	3	90.0	0.133	(−0.663 to 0.930)	0.743
Retrospective	8	81.8	0.169	(−0.040 to 0.378)	0.120
Mortality rate					
More than 60%	3	89.8	0.044	(−0.692 to 0.781)	0.906
30–60%	3	1.1	0.060	(−0.085 to 0.205)	0.418
Up to 30%	5	77.3	0.321	(0.043 to 0.598)	0.023

SMD: standardized mean difference.

**Table 3:** Sensitivity analysis of possible heterogeneity studies.

Reason of exclusion	Excluded studies	Number of studies	*I* square (%)	SMD	(95% confidence interval)	*p* value
Lowest mortality	Kitazawa et al. [20]	10	83.5	0.197	(−0.020 to 0.414)	0.076
Highest mortality	Gao et al. [22]	10	82.6	0.114	(−0.098 to 0.326)	0.293
Lowest and highest mortality	Kitazawa et al. [20]	9	83.4	0.139	(−0.084 to 0.361)	0.221
	Gao et al. [22]					
Small studies (*N* < 100)	Becchi et al. [18]	9	83.5	0.241	(0.023 to 0.030)	0.030
	Zampieri et al. [24]					
