# Supplementary material for: The Role of Mean Platelet Volume as a Predictor of Mortality in Critically Ill Patients: A Systematic Review and Meta-Analysis
Source: Crit Care Res Pract. 2016 Feb 4;2016:4370834. doi: 10.1155/2016/4370834 (PMC4757676; doi:10.1155/2016/4370834)
Supplement: Supplementary file 1 — Search terms and search strategy used for PubMed, Scopus and Embase (“mean platelet volume” OR “platelet ind∗” OR “platelet size∗”) AND (sepsis OR “septic shock” OR “intensive care” OR “critically ill∗”). [file 4370834.f1.pdf]

Supplement Appendix

Appendix A. Search terms and search strategy used for PubMed, Scopus and Embase

("mean platelet volume" OR "platelet ind*" OR "platelet size*") AND (sepsis OR "septic shock" OR "intensive care" OR "critically ill*")

Supplemental figures 1–11

Supplement figure 1. The pooled mean differences of the mean platelet volume between critically ill non-survivors and survivors: subgroup analysis by ***"patient setting (sepsis vs. all ICU)"***.

Supplement figure 2. The pooled mean differences of the mean platelet volume between critically ill non-survivors and survivors: subgroup analysis by "***type of study design"***.

Supplement figure 3. The pooled mean differences of the mean platelet volume between critically ill non-survivors and survivors: subgroup analysis by ***"mortality rate more than 60 percent, 30 – 60 percent and up to 30 percent***".

Supplement figure 4. The pooled mean differences of the mean platelet volume between critically ill non-survivors and survivors: sensitivity analysis by ***exclusion the small studies (total sample less than 100)***.

Supplement figure 5. The pooled mean differences of the mean platelet volume between critically ill non-survivors and survivors on "***the first day***".

Supplement figure 6. The pooled mean differences of the mean platelet volume between critically ill non-survivors and survivors on ***"the second day".***

Supplement figure 7. The pooled mean differences of the mean platelet volume between critically ill non-survivors and survivors on "***the third day"***.

Supplement figure 8. The pooled mean differences of the mean platelet volume between critically ill non-survivors and survivors on "***the fourth day".***

Supplement figure 9. The pooled mean differences of the mean platelet volume between critically ill non-survivors and survivors on "***the fifth day***".

Supplement figure 10. The pooled mean differences of the mean platelet volume between critically ill non-survivors and survivors on "***the seventh day"***.

Supplement figure 11: The funnel plot of the mean differences in the mean platelet volume between survivors and non-survivors among critically ill patients.

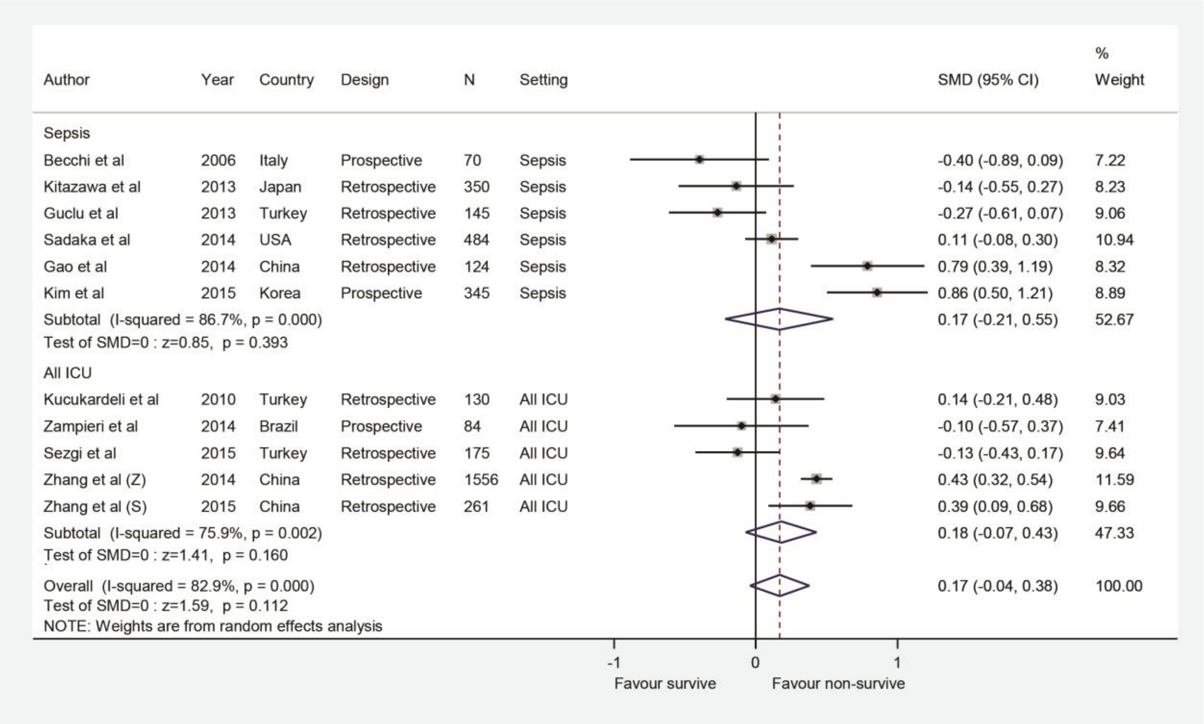

Supplement figure 1. The pooled mean differences of the mean platelet volume between critically ill non-survivors and survivors: subgroup analysis by ***"patient setting (sepsis vs. all ICU)"***.

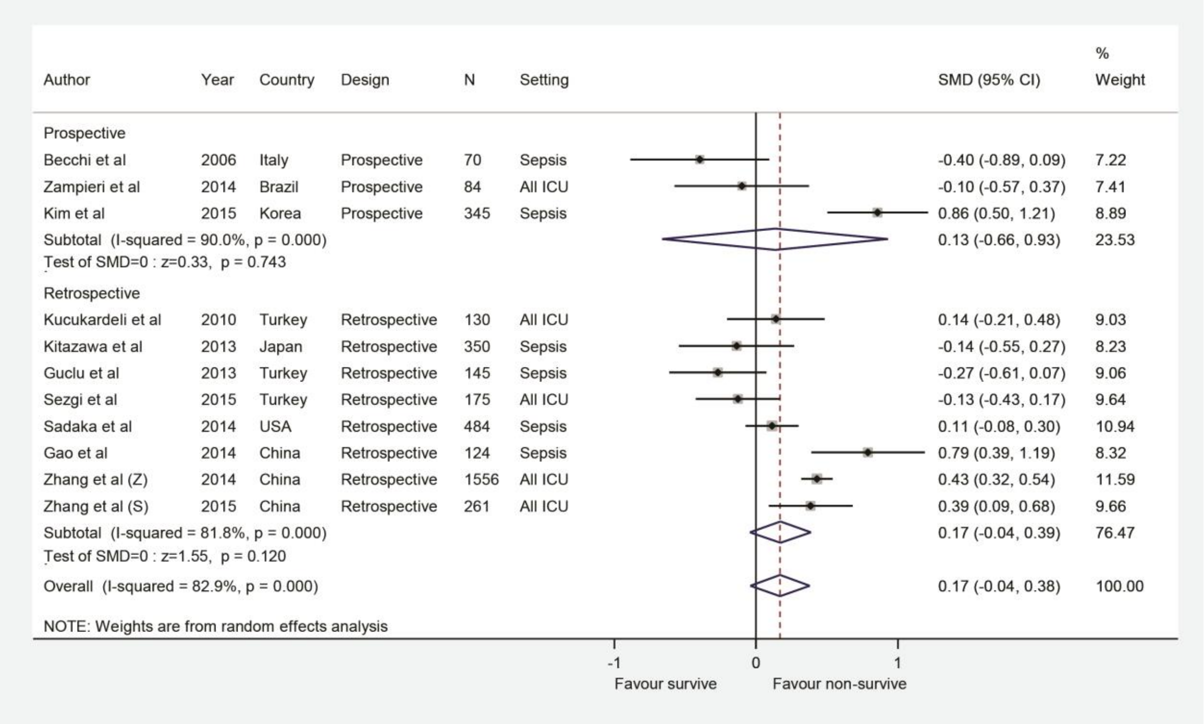

Supplement figure 2. The pooled mean differences of the mean platelet volume between critically ill non-survivors and survivors: subgroup analysis by "***type of study design"***.

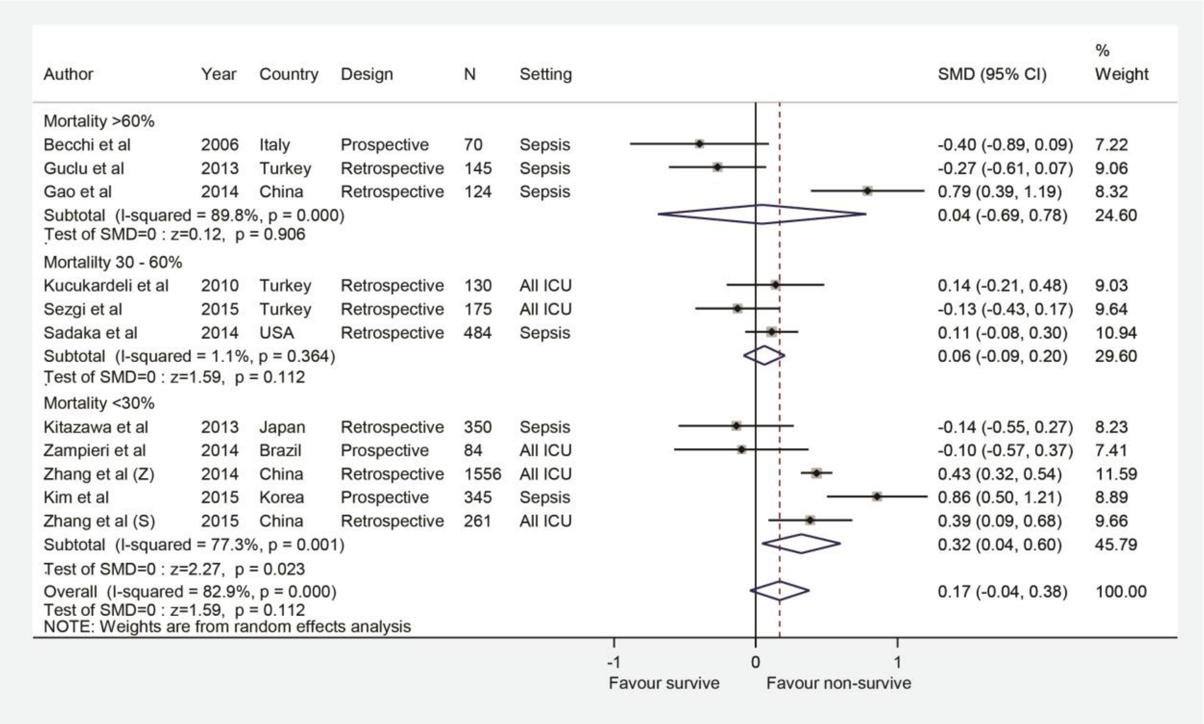

Supplement figure 3. The pooled mean differences of the mean platelet volume between critically ill non-survivors and survivors: subgroup analysis by ***"mortality rate more than 60 percent, 30 – 60 percent and up to 30 percent***".

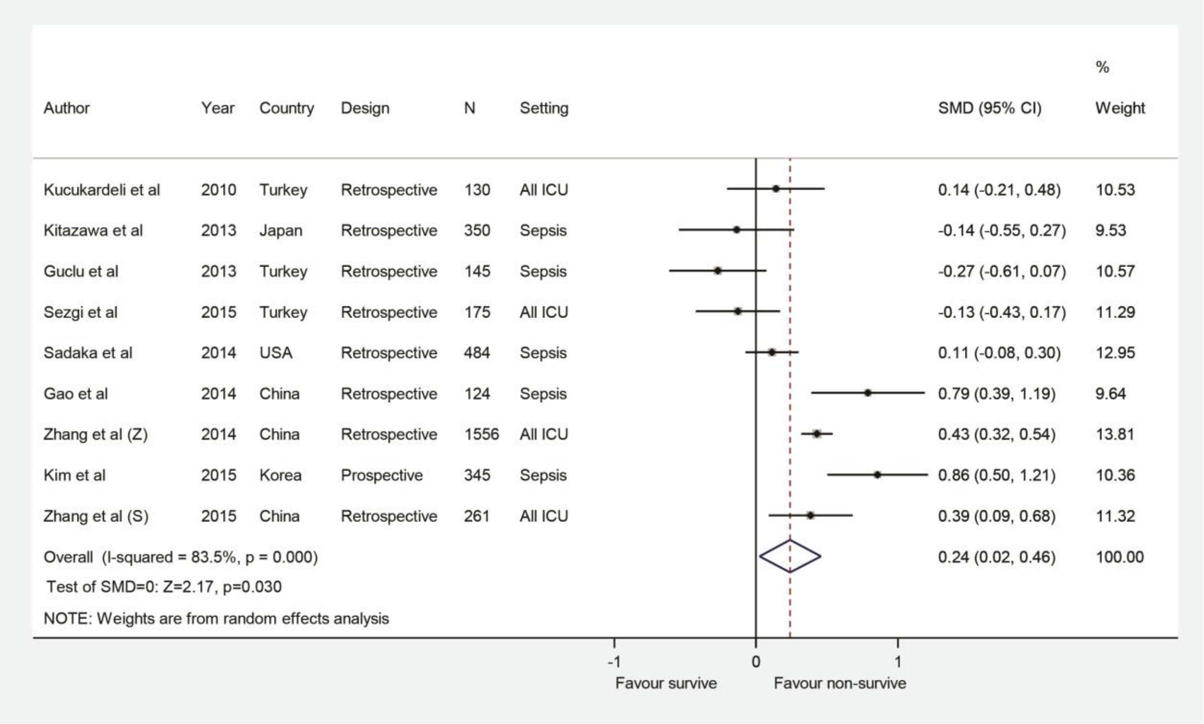

Supplement figure 4. The pooled mean differences of the mean platelet volume between critically ill non-survivors and survivors: sensitivity analysis by ***exclusion the small studies (total sample less than 100)***.

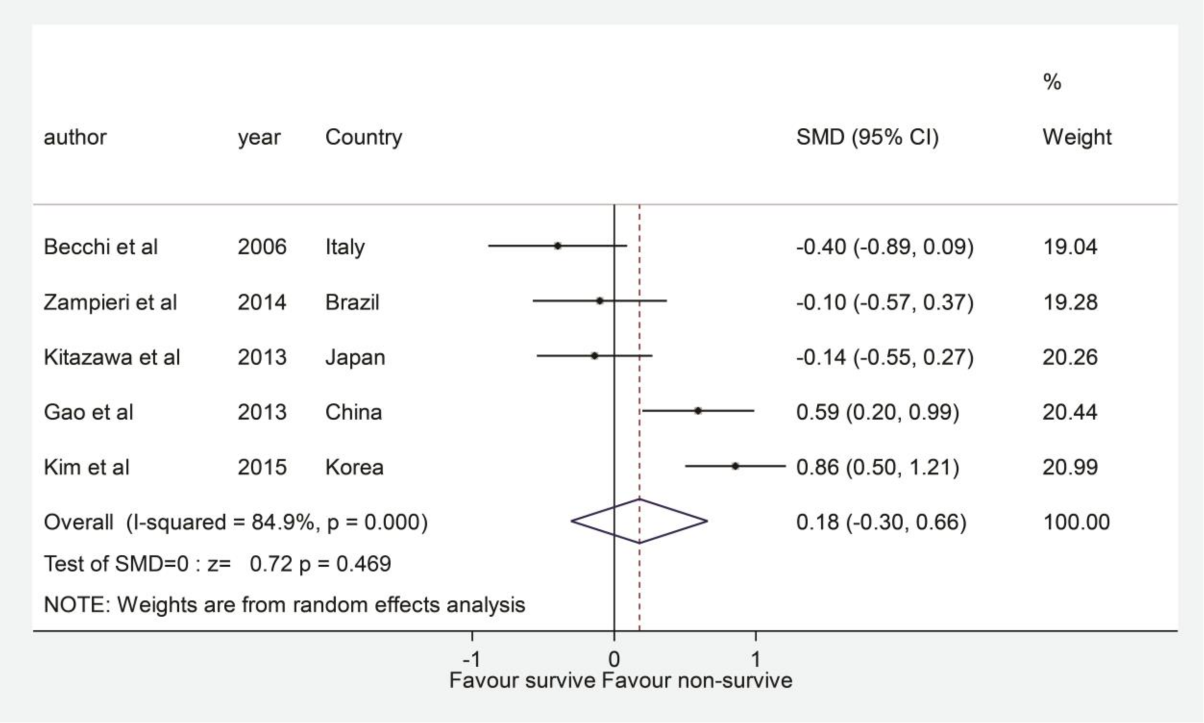

Supplement figure 5. The pooled mean differences of the mean platelet volume between critically ill non-survivors and survivors on "***the first day***".

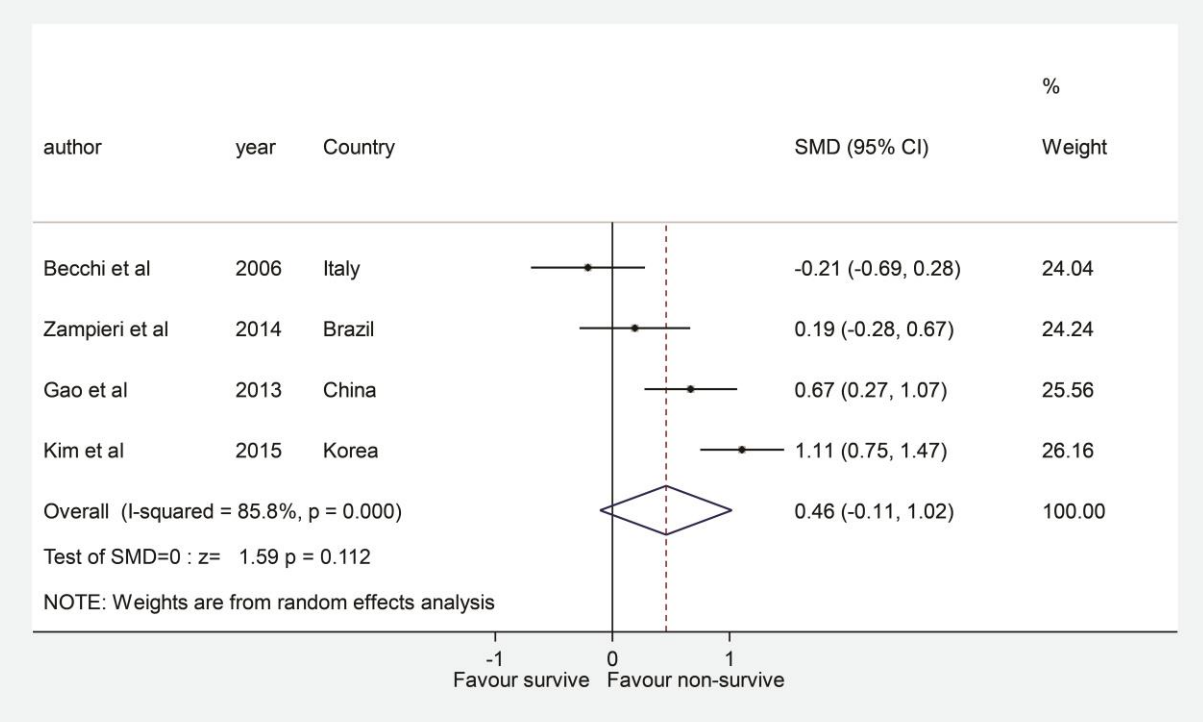

Supplement figure 6. The pooled mean differences of the mean platelet volume between critically ill non-survivors and survivors on ***"the second day".***

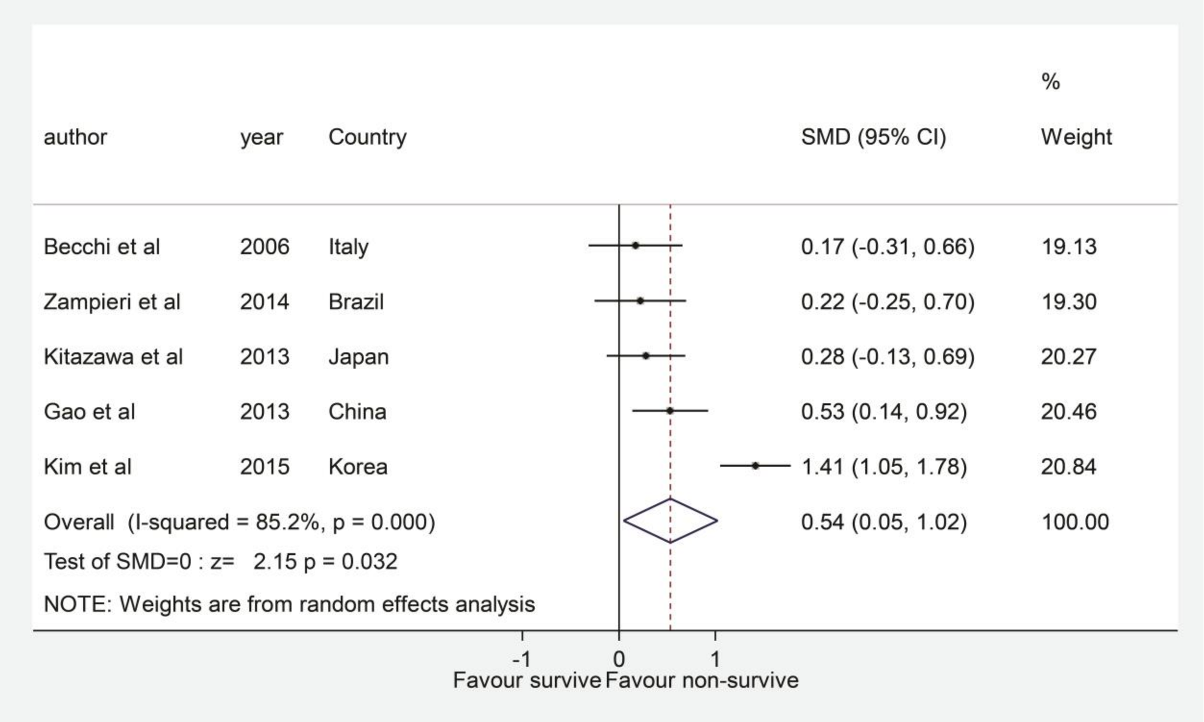

Supplement figure 7. The pooled mean differences of the mean platelet volume between critically ill non-survivors and survivors on “***the third day***”.

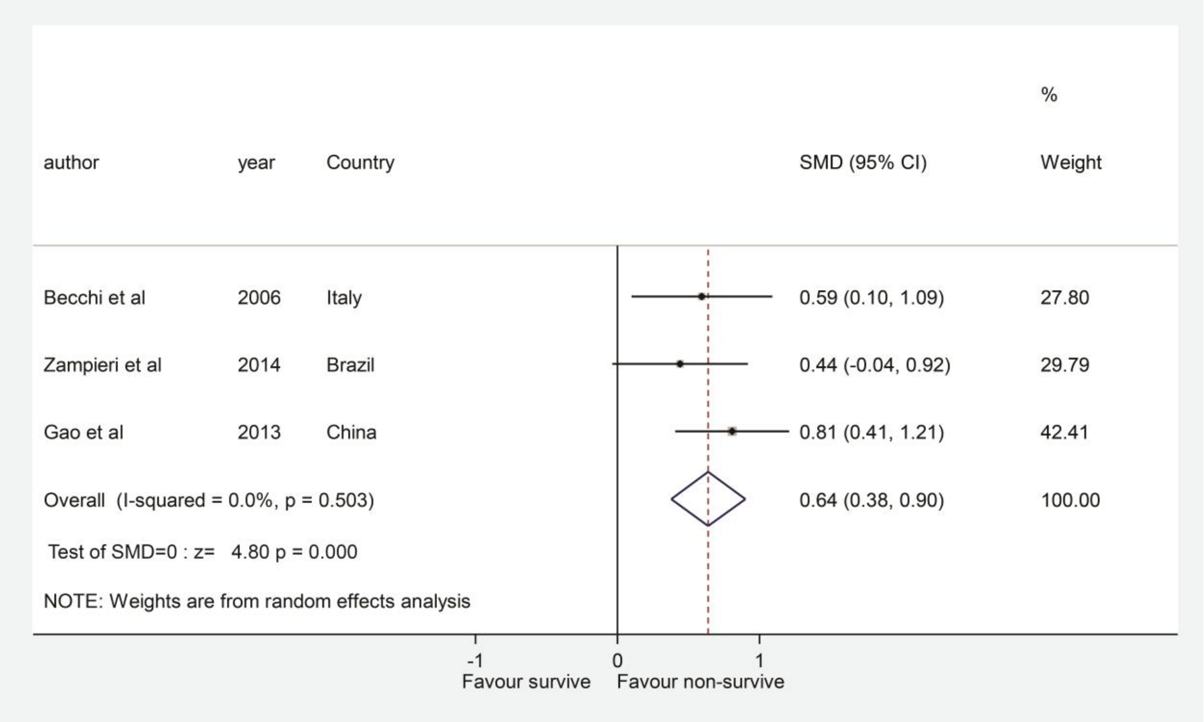

Supplement figure 8. The pooled mean differences of the mean platelet volume between critically ill non-survivors and survivors on “***the fourth day***”.

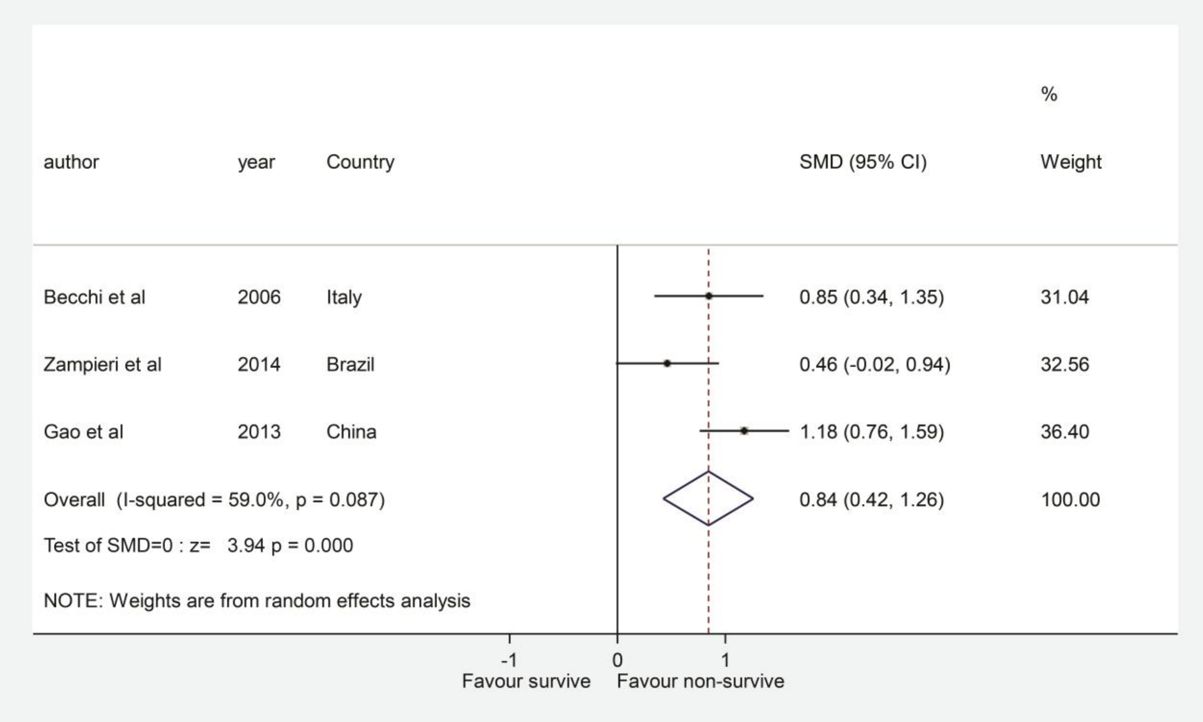

Supplement figure 9. The pooled mean differences of the mean platelet volume between critically ill non-survivors and survivors on "***the fifth day***".

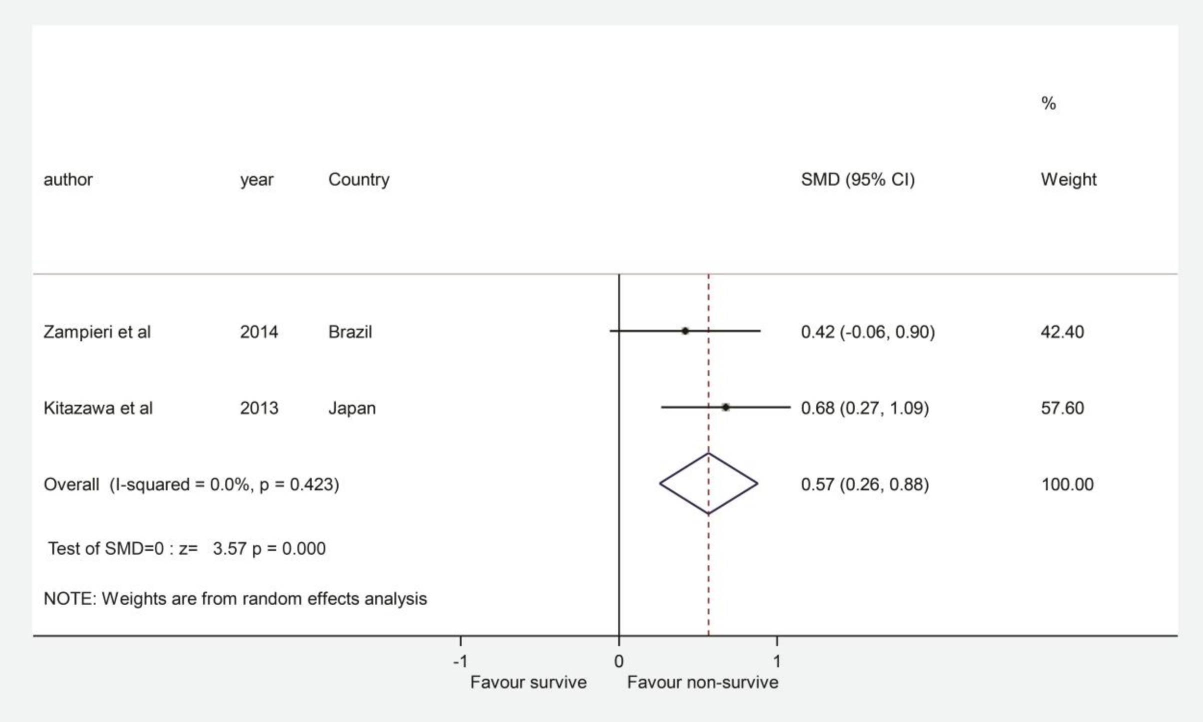

Supplement figure 10. The pooled mean differences of the mean platelet volume between critically ill non-survivors and survivors on "***the seventh day***".

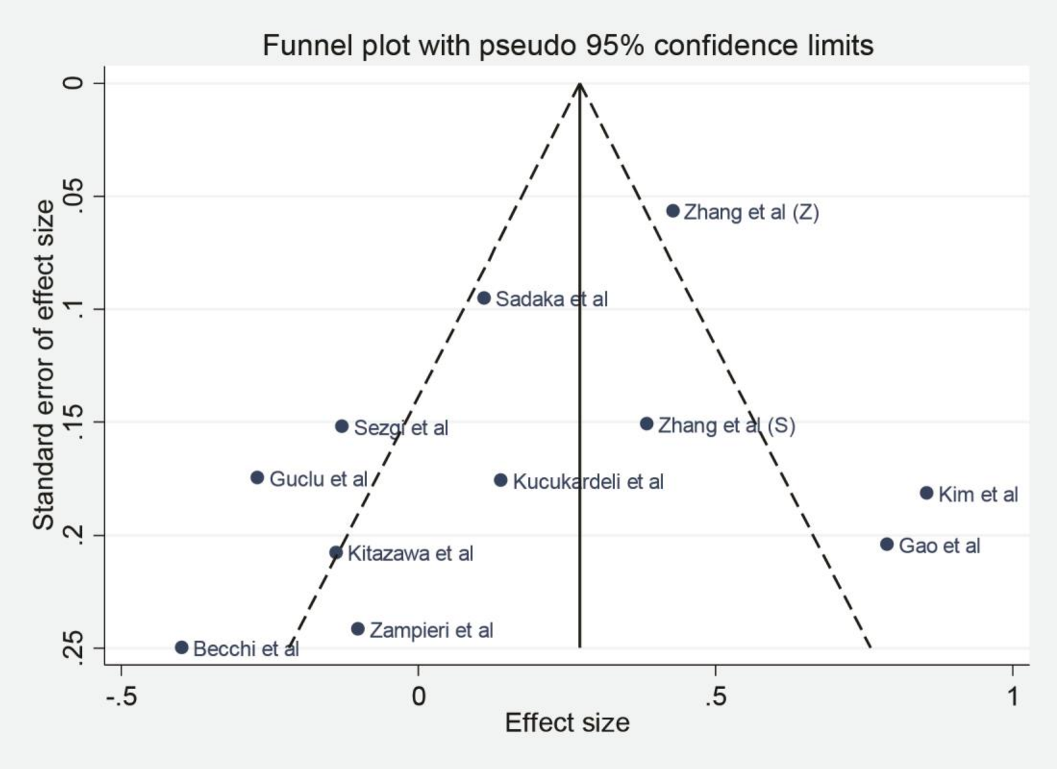

Supplement figure 11: The funnel plot of the mean differences in the mean platelet volume between survivors and non-survivors among critically ill patients.

Supplemental tables 1–3

Supplemental table 1: Daily MPV in Survivors and Non-survivors among Critically Ill Patients

Supplemental table 2: Risk of Bias Assessment for Cohort Studies

Supplemental table 3: meta-regression on the possible sources of heterogeneity based on study characters

Supplemental table 1: Daily MPV in Survivors and Non-survivors among Critically Ill Patients

Author	Year		MPV within day 1	MPV within day 2	MPV within day 3	MPV within day 4	MPV within day 5	MPV within day 7
Becchi et al. [18]	2006	Non-survivors	9.96 ± 1.70	10.15 ± 1.70	10.47 ± 1.80	10.80 ± 1.50	11.10 ± 1.60	-
		Survivors	10.54 ± 0.90	10.45 ± 0.80	10.20 ± 1.10	10.02 ± 0.90	9.88 ± 1.10	-
Kitazawa et al. [20]*	2013	Non-survivors	7.47 ± 1.06	-	8.13 ± 2.04	-	-	8.35 ± 1.88
		Survivors	7.61 ± 1.01	-	7.80 ± 1.09	-	-	7.57 ± 1.08
Zampieri et al. [24]	2014	Non-survivors	10.80 ± 0.85	11.09 ± 0.96	11.20 ± 0.92	11.35 ± 0.97	11.31 ± 0.92	11.23 ± 0.81
		Survivors	10.90 ± 1.04	10.90 ± 1.0	10.97 ± 1.08	10.89 ± 1.07	10.84 ± 1.05	10.78 ± 1.16
Gao et al. [22] **	2014	Non-survivors	11.0 (10.3, 12.1)	10.9 (10.4, 12.0)	11.1 (10.5, 12.0)	11.5 (10.4, 12.6)	12.1 (10.8, 12.9)	-
		Survivors	10.3 (10.0, 10.9)	10.4 (10.0, 10.8)	10.4 (9.9, 11.5)	10.2 (9.7, 11.1)	10.0 (9.6, 11.1)	-
Kim et al.[26] ***	2015	Non-survivors	9.54 ± 1.66	9.96 ± 1.76	10.35 ± 1.69	-	-	-
		Survivors	8.54 ± 1.10	8.65 ± 1.10	8.80 ± 1.01	-	-	-

Note: The data are shown as mean ± SD and median (IQR).

*Kitazawa et al reported MPV by the time period 0–1 day, 3–5 days, and 7–10 days, respectively. We assumed the period “0–1” as within day 1, the period “3–5” as within day 3, and the period “7–10” as within day 7.

**The daily MPV data were obtained from “Change of platelet parameters in septic shock patients” published in Chinese critical care medicine, January 2014, Volume 26 (1): 28-32.

***Chan et al reported MPV at baseline, 36 hours, and 72 hours; hence, we assumed the MPV value at 36 hours as MPV within day 2.

Supplemental table 2: Risk of Bias Assessment for Cohort Studies

Author	Year	Selection				Comparability	Outcome			Total score
		**Representativeness of cohort**	**Selection of non-exposed cohort**	**Ascertainment of exposure**	**Outcome of interest**	**Comparability of cohorts**	**Assessment of outcome**	**Adequate duration of follow-up**	**Adequate follow-up of cohort**	
Becchi et al. [18]	2006	B (1*)	A (1*)	A (1*)	A (1*)	0	A (1*)	A (1*)	D	6
Kucukardeli et al. [17]	2010	B (1*)	A (1*)	A (1*)	A (1*)	0	A (1*)	A (1*)	D	6
Guclu et al.[19]	2013	A (1*)	B (1*)	A (1*)	A (1*)	0	A (1*)	A (1*)	D	6
Kitazawa et al.[20]	2013	B (1*)	A (1*)	A (1*)	A (1*)	A (1*)	A (1*)	A (1*)	A (1*)	8
Sadaka et al.[21]	2014	A (1*)	A (1*)	A (1*)	A (1*)	0	A (1*)	A (1*)	D	6
Zampieri et al.[24]	2014	B (1*)	A (1*)	A (1*)	A (1*)	0	A (1*)	A (1*)	A (1*)	7
Sezgi et al.[23]	2015	C (0)	A (1*)	A (1*)	A (1*)	0	A (1*)	A (1*)	D	5
Zhang et al.(Z)[25]	2014	B (1*)	A (1*)	A (1*)	A (1*)	0	A (1*)	A (1*)	A (1*)	7
Gao et al.[22]	2014	B (1*)	A (1*)	A (1*)	A (1*)	0	A (1*)	A (1*)	D	6
Kim et al.[26]	2015	B (1*)	A (1*)	A (1*)	A (1*)	0	A (1*)	A (1*)	A (1*)	7
Zhang et al. (S)[27]	2015	B (1*)	A (1*)	A (1*)	A (1*)	0	A (1*)	A (1*)	D	6

*The Newcastle and Ottawa risk of bias criteria score, A–D are the risk of bias criteria classification of the Newcastle and Ottawa method.

Supplemental table 3: meta-regression on the possible sources of heterogeneity based on study characters

Possible sources of heterogeneity	Number of studies	I square (%)	p value
All	11	82.9	0.196
Study design*	11	84.6	0.995
ICU setting**	11	83.4	0.989
Mortality rate***	11	78.7	0.353
Age in non-survive	10	83.7	0.940
Age in survive	10	82.6	0.660
Male in non-survive	7	72.0	0.087
Male in survive	7	85.4	0.644
APACHE II score in non-survive	6	85.5	0.755
APACHE II score in survive	6	85.4	0.834
SOFA score in non-survive	4	87.6	0.546
SOFA score in survive	4	87.0	0.797

*Prospective study vs. Retrospective study; **Sepsis vs. All ICU patients; ***Mortality rate <30% vs.30-60% vs.>60%
